# Organoids: a promising new in vitro platform in livestock and veterinary research

**DOI:** 10.1186/s13567-021-00904-2

**Published:** 2021-03-10

**Authors:** Soumya K. Kar, Jerry M. Wells, Esther D. Ellen, Marinus F. W. te Pas, Ole Madsen, Martien A. M. Groenen, Henri Woelders

**Affiliations:** 1 Wageningen Livestock Research, Wageningen University & Research, Wageningen, The Netherlands; 2 Host-Microbe Interactomics, Wageningen University & Research, Wageningen, The Netherlands; 3 Animal Breeding and Genomics, Wageningen University & Research, Wageningen, The Netherlands

**Keywords:** Animal health, Animal breeding and genomics, Animal nutrition, Host-microbe interaction, In vitro model, Organoids, Stem cell research, Veterinary research

## Abstract

Organoids are self-organizing, self-renewing three-dimensional cellular structures that resemble organs in structure and function. They can be derived from adult stem cells, embryonic stem cells, or induced pluripotent stem cells. They contain most of the relevant cell types with a topology and cell-to-cell interactions resembling that of the in vivo tissue. The widespread and increasing adoption of organoid-based technologies in human biomedical research is testament to their enormous potential in basic, translational- and applied-research. In a similar fashion there appear to be ample possibilities for research applications of organoids from livestock and companion animals. Furthermore, organoids as in vitro models offer a great possibility to reduce the use of experimental animals. Here, we provide an overview of studies on organoids in livestock and companion animal species, with focus on the methods developed for organoids from a variety of tissues/organs from various animal species and on the applications in veterinary research. Current limitations, and ongoing research to address these limitations, are discussed. Further, we elaborate on a number of fields of research in animal nutrition, host-microbe interactions, animal breeding and genomics, and animal biotechnology, in which organoids may have great potential as an in vitro research tool.

## Introduction

In the first decade of the present century, it was shown that stem cells grown in vitro with required growth and differentiation factors in the presence of extracellular matrix (ECM) components can proliferate while maintaining “stemness”, the ability to self-renew and give rise to self-organizing three dimensional (3D) structures [1, 2]. While the term “organoids” has been used in the literature for decades, the feature of stem cell-based self-renewal and self-organization of multicellular (3D) structures containing multiple organ-specific cell in a manner similar to in vivo is used to define “organoids” in most current studies [3, 4]. Organoids can be generated from adult stem cells (ASCs) [2]; embryonic stem cells (ESCs) [1]; or from induced pluripotent stem cells (iPSCs), i.e. stem cells generated by “reprogramming” differentiated somatic cells (e.g. skin fibroblasts) to regain pluripotency [5, 6]. This review will focus on stem cell-derived self-organizing and self-renewing organoids, as defined by Lancaster and Knoblich [4]. Moreover, we will briefly mention other studies on 3D cell structures that do not feature stem cell-based self-renewal, and also do not demonstrate the recent criteria defining the term organoid [4].

Organoid cultures can typically be maintained for very long times (months, or even longer than a year), as shown for organoids derived from e.g. intestine [2], stomach [7], liver [8], and pancreas [9], as well as for iPSC-derived [10] organoids and ESC-derived [11] organoids. Organoid cultures can remain committed to their tissue of origin and capable of recapitulating the pathology of disease when cultured with tissues derived from clinical patients [12, 13]. Moreover, organoids can be easily cryopreserved [14] and cultures can be restored from cryopreserved stocks, retaining functionality similar to that of the tissue of origin. They are amenable to genetic manipulation [15], live imaging, gene expression analysis, sequencing and epigenetic analysis, and other standard biological analyses. Organoids contain multiple cell types, with tissue topology and cell–cell interaction resembling many key features of the in vivo organ or tissue [16] whereas cell lines are usually derived from tumors or have become cancerous in vitro. Cancer-derived cell lines typically possess chromosomal aberrations and mutations [17] affecting growth, metabolism and physiology and are known to evolve during continuous passage in vitro creating problems with reproducibility [18]. Organoids also have an advantage over tissue explants or primary cell cultures, which undergo senescence, cell death and necrosis over relatively short time spans, leading to poor reproducibility and accuracy of biological experiments. These qualities have led to an exponentially increasing interest in this field during the last decade [3, 4, 19], and organoid technology was announced as one of the biggest scientific advancements of 2013 by The Scientist [20] and “Method of the Year 2017” in the Nature Methods editorial [21].

As organoids can be derived from cells or tissue from individual humans or animals, they can be used for testing patient-specific drug response [22] or patient-specific–autologous–grafting of genetically “repaired” tissues [23, 24]. For farm animals, animal-specific organoids could potentially be used for in vitro phenotyping, testing in vitro characteristics of organoids that may be a proxy for traits of interest [25, 26].

In human biomedical research, organoids are finding broad applications as in vitro research models, for instance for studying toxicology, developmental processes, congenital diseases, infectious diseases [27, 28], cancer [29, 30] and in regenerative medicine [31].

**Figure 1:**
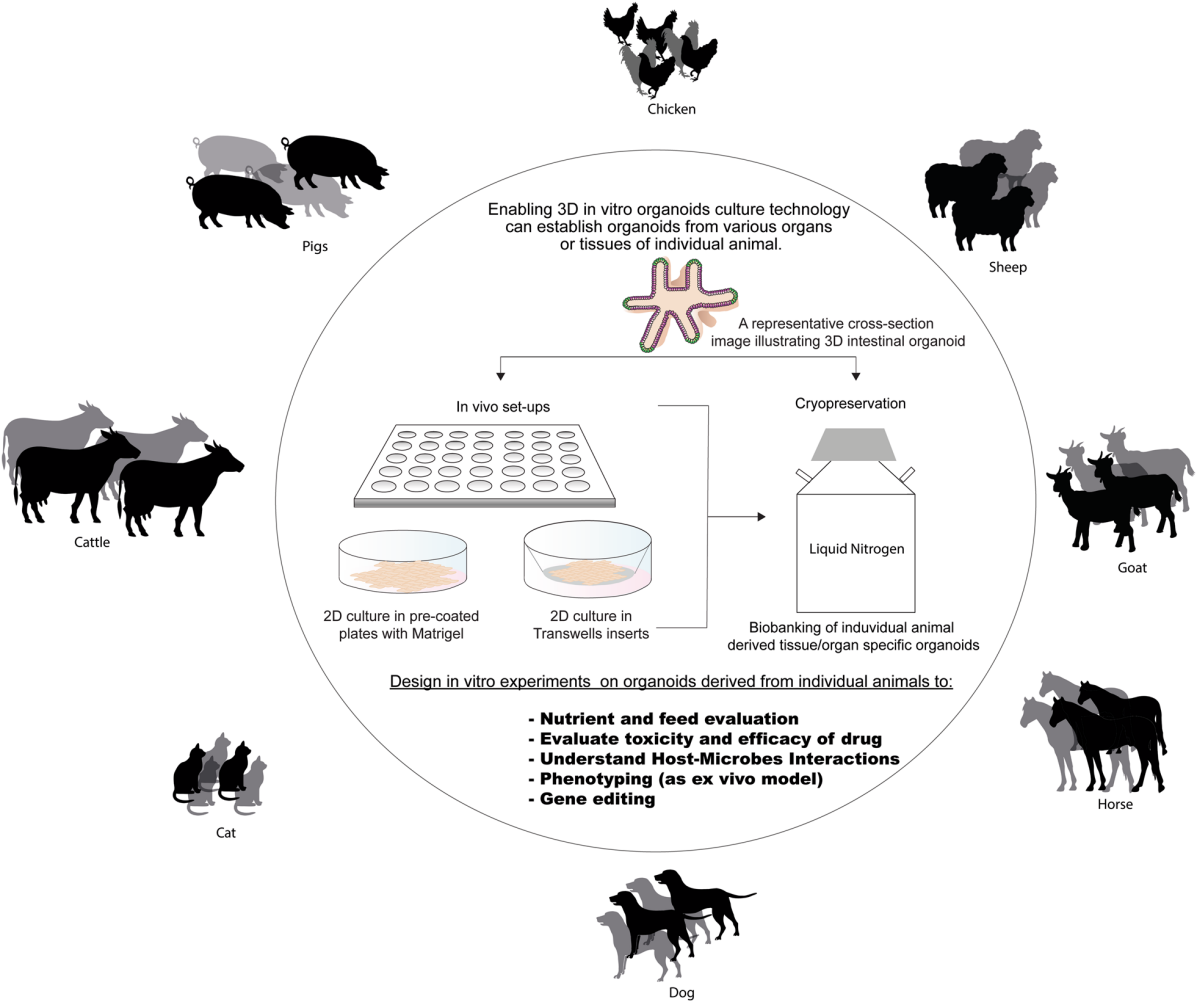
**Application opportunities of organoids in livestock and veterinary research.**

In contrast, there have been relatively few studies on organoids in veterinary and animal production research, despite the potential application and impact in research on animal physiology, animal nutrition, host-microbe interaction (HMI), and for in vitro phenotyping of breeding animals. In this paper, we focus on organoid research in livestock and companion animals, reviewing the used methodologies, applications, and future prospective for organoid research in livestock and companion animals (Figure 1).

## Organoid derivation and culture methods

**Figure 2:**
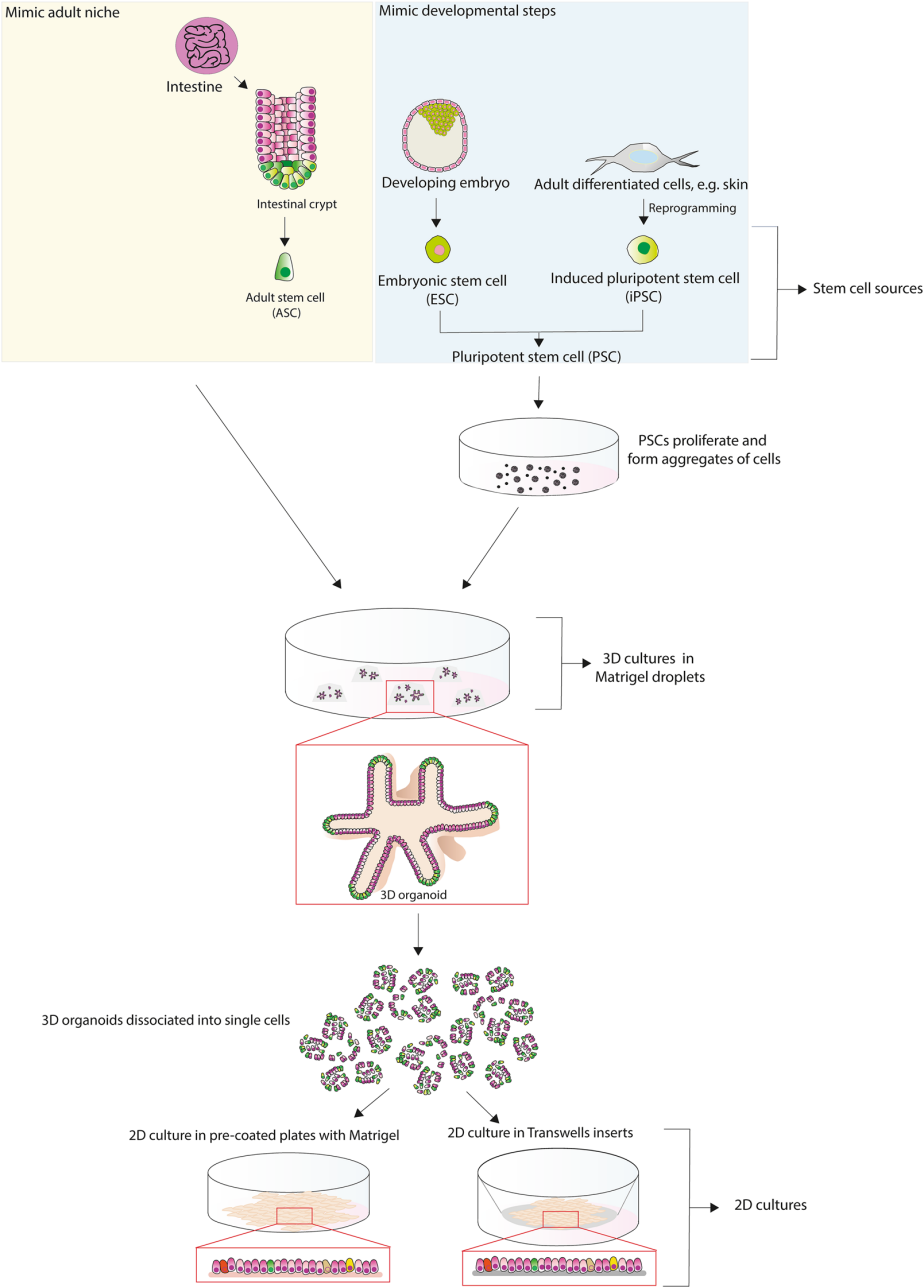
**Current organoid culture techniques.** Organoids can be derived from tissue samples containing adult stem cells (ASCs). Here, intestinal crypts are shown as example, but methods for other tissues (see main text) are similar. Organoids can also be derived from embryonic stem cells (ESCs), or induced pluripotent stem cells (iPSCs). Under appropriate conditions, using various growth factors and an extracellular matrix, such as matrigel (MG), the stem cells can proliferate while their daughter cells can differentiate to multiple cell types that self-organize into functional three dimensional (3D) structures. Different tissues require different (combinations of) growth factors. The 3D organoids can be dissociated, and plated onto membrane supports coated with MG or collagen, to form 2D monolayer organoid models. This is particularly useful of intestinal organoids as it allows access to the apical side, for instance to study interaction with microbes, or transport of nutrients.

Organoids can be generated from ASCs, ESCs, or iPSCs (Figure 2). ESCs are the stem cells from the inner cell mass of pre-implantation embryos. ASCs in principle are obtained from “mature” or adult tissue, but this is not necessarily tissue from adult animals but may be from juveniles or even from advanced embryos [3, 4]. ASC-derived organoids are intrinsically programmed with their location-specific functions [32], making them more “adult-like” than organoids derived from iPSC or ESC, although the latter retain tissue-associated mesenchymal cells [33].

Under appropriate conditions, stem cells can divide to give rise to one daughter cell that maintains “stemness”, while the other daughter cell can differentiate to a dedicated tissue-specific cell type. In tissues in vivo, the direct microenvironment, or “niche” of the stem cells and the differentiating daughter cells provide various signals that direct these processes and steer the direction of differentiation and thus determine what types of cells and tissue will develop. These signals include cell-to-cell contacts [34], contacts of cells to compounds of the ECM, autocrine growth factors and hormones from neighboring cells, from cells elsewhere in the tissue, or from peripheral organs [4]. The 3D architecture itself is instrumental in directing the spatial organization of cell lineages, as it creates gradients of growth factors determining the specific cellular differentiation steps [35].

The use of 3D culture matrices for organoids exploit the mechanisms that steer development of the cells in vivo. Therefore, the knowledge gained from stem cell biology and the insights obtained through 3D in vitro culturing methodology have been crucial in developing methods for generating organoid models for a multitude of organs [36].

Matrigel^®^ (MG), a de-cellularized ECM secreted by Engelbreth-Holm-Swarm mouse sarcoma cells [37], is typically used as matrix for 3D culture. Alternative ECMs include the synthetic hydrogel matrices [38]. Matrigel^®^ provides specific signals conferred from the binding of cells to ECM components like ECM-proteins e.g. laminin, collagen IV, entactin/nidogen, heparan sulfate proteoglycans. Furthermore, it provides ECM-associated-growth factors like IGFs, FGFs, TGF-beta’s, and HGF [39]. Moreover, the ECM density, stiffness, viscoelasticity, as well as topology and fibrosity are key ECM parameters that govern stem cell expansion and organoid formation [40].

In the case of epithelial organoids, the 3D structure can be a disadvantage for practical reasons. For instance, 3D intestinal organoids feature a miniature internal “lumen”, very much like a true intestine, making it difficult to access the apical (luminal) side for studying interaction with microbes or trans-epithelial nutrient transport. For that reason, methods have been developed to generate two-dimensional (2D) polarized epithelial monolayers by seeding dissociated cells of 3D organoids onto MG or collagen-coated Transwell membranes [41–43], as described in more detail in Sect. 4.

## Research in major livestock and companion animals

### Tissue-explants and re-aggregated dissociated cells

In this section, we only briefly touch on studies on “organoid-like” 3D cultures that have used tissue explants and/or re-aggregated dissociated tissue cells, (Table 1). Many of these studies are focused on tissue engineering for repair or replacement, or for extracorporeal artificial organ devices, rather than on developing in vitro research models. However, the 3D cultures in some of the earlier papers listed in Table 1 do seem to share certain features with self-renewing organoids, describing differentiation of organ-like structures from progenitor cells using ECM components and growth factors to induce 3D tissue development.

**Table 1:** Studies on tissue explants and re-aggregated dissociated cells in livestock and companion species

Species	Tissue	References
Pigs	Intestine & stomach	[145]
	Bile duct	[146]
	Liver	[147–151]
	Urinary bladder	[152]
	Testis	[44, 45]
Chicken	Nervous and lymphoid tissue	[47]
	Skeletal muscles	[46]
Cattle	Arteries	[153–157]
	Articular cartilage	[158, 159]
	Intestine	[160]
	Mammary gland	[48–52]
	Parathyroid gland	[161–166]
Dog	Intestine	[167]

Two studies reported the production of porcine testicular “organoids” from dissociated testis [44, 45]. The developed testicular structures contained all relevant cell types in a 3D organization with physiological cell–cell interactions of germ cells with supporting cells [44]. In addition, the cultured testicular structures contained Sertoli cells and germ cells which assembled into seminiferous tubule-like structures delimited by a basement membrane along with Leydig cells and peritubular cells. In one of these studies, the cellular structures were maintained for 45 days [45]. However, long term self-renewal was not well-controlled, as the number of Sertoli cells increased and that of Leydig cells decreased over time. Yet, these organoids appear to provide an in vitro platform for studying germ cell function, testicular development, and drug toxicity in a cellular context representative of the testis in vivo.

In chicken, two early studies showed long term culture of 3D organ-like structures (“organoids”) of chicken muscle [46] and nervous and lymphoid tissues [47], respectively. These studies used ECM (MG or collagen) and certain growth factors, but these models lacked continuous stem cell-driven proliferation.

In cattle, Ellis et al. reported long-term culture of bovine 3D mammary gland organoids that recapitulated glandular duct morphology and function [48]. Their culture methods included the use of ECM (collagen) and growth factors, such as IGF1, TGFβ, and mammary gland extract. Similar methods were used by other authors [49–52].

### Stem cell-derived organoids in livestock and companion animals

Stem cell-derived organoids that are capable of self-renewal and self-organization in long-term culture (Figure 2), are listed in Table 2. In all these studies, organoids were derived from tissue ASCs. To our knowledge no published scientific studies are available on iPSC-derived organoids in farm or companion animals.

**Table 2:** Summary of livestock and companion species organoid

Species	Tissue	References
Pig	Esophagus submucosal gland	[58]
	Intestine	[43, 53–56, 59–64, 71–74, 76–81]
	Rectum	[57]
Chicken	Intestine	[55, 82–87]
Cattle	Intestine	[55, 56, 88, 89]
Sheep	Intestine	[55]
	Pancreatic duct	[90]
Dog	Skin (Keratinocyte)	[93]
	Prostate gland	[98]
	Urinary bladder	[99]
	Kidney	[97]
	Intestine	[55, 91]
	Liver	[96]
Cat	Intestine	[55, 100]
	Liver	[101, 102]
Horse	Intestine	[55, 103]

#### Pig

Intestinal organoids (enteroids) were the first organoids reported from pigs. Gonzalez et al. demonstrated development of enteroids in vitro from intestinal crypts from new-born piglets [53]. These enteroids showed budding (forming buds with a crypt-like structure) and contained the principal cell types of the intestinal epithelium including stem/progenitor cells, absorptive enterocytes, enteroendocrine cells, goblet cells, and Paneth-like cells. Later studies applied similar methods with small modifications that were necessary for, or contributed to, the development of enteroids from juvenile and adult pig intestinal tissue [54], or the ability to maintain the enteroids in culture for several months [43, 55, 56]. Organoids have also been derived successfully from the rectum [57], esophageal submucosal gland [58], and colon (often referred to as “colonoids”) [59]. A recent study showed that differential gene- and pathway expression of independent organoid cultures from the same pig was stable over 12 weeks of culture [60]. Furthermore the batch-to-batch variation in organoid gene expression was low during long term culture, which may have aided by simultaneous passage to limit differences in their differentiation state. Moreover, in the same study, the authors also compared the transcriptomes profiles of jejunal organoids, the corresponding jejunum mucosa tissue from which the organoids were derived, and IPEC-J2 cells (a porcine cell line derived from the jejunum, often used as model for small intestinal epithelium). Below in Sect. 4.4, the same study is mentioned as “validation” in the lights that organoids are compared with tissue and IPEC-J2 cells.

Pig intestinal organoids have been applied to study nutrition, nutrient uptake, feed efficiency, and interaction with pathogenic microbes (viruses, bacteria). Koltes and Gabler have applied porcine intestinal organoids to study LPS-induced intestinal inflammation in pigs [61]. Likewise, Derricott et al. also produced and characterized murine, bovine, and porcine enteroids as potential research models for the study of species-specific intestinal infections with a variety of pathogens [56]. They demonstrated in vitro infection of bovine and porcine enteroids by the eukaryotic parasite *Toxoplasma gondii* and the bacterial pathogen *Salmonella typhimurium*. Resende et al. showed that *Lawsonia intracellularis* is capable of infecting and replicating intracellularly in 2D pig enteroids, which resulted in epithelial changes as observed in the *Lawsonia*-infected enteroids, specifically regarding the intestinal cell constitution and gene expression [62]. Further, a pig enteroid model was used to study porcine epidemic diarrhea virus (PEDV) infection [63, 64]. The identity of the specific cell types targeted by PEDV in vivo has remained elusive. Most in vitro studies on PEDV infection have been performed in cell lines of other than pig origin, such as Vero cells from African green monkey kidney and HEK293 cells from human embryonic kidney [65–67]. Vero cells are incapable of producing type I interferons when infected by viruses [68]. PEDV clinical isolates generally do not replicate very well in IPEC-J2 cells either [69, 70]. In contrast, PEDV was shown to infect multiple types of epithelial cells of a porcine enteroid model, including enterocytes, stem cells, and goblet cells [63, 64]. These studies also provided insights into the porcine interferon defense mechanisms. Furthermore, pig enteroid model has been employed to study Porcine deltacoronavirus (PDCoV) tropism to different intestinal segments [71] and the molecular mechanisms of PDCoV infection [72]. Recently, Li et al. reported to have developed a porcine apical-out intestinal organoid culture system and verified its infectivity, type I and type III interferon (IFN) antiviral responses, and inflammatory responses following infection by a swine enteric virus i.e. by transmissible gastroenteritis virus [73]. Overall, the above cited studies on the interaction of pathogens with intestinal epithelia clearly shows the suitability of enteroids and colonoids as in vitro intestinal models to study host–pathogen interaction in pigs.

At the interface of nutritional and immune research, Ellen et al. used porcine enteroids and colonoids to study host–pathogen interaction in relation to feed efficiency [59]. Ferrandis et al. used porcine and murine enteroids to study the role of cytokines (like interleukins (IL)-1β and IL-4) in the regulation of mucin production (i.e. expression of the MUC2 gene) by the epithelium, as dietary fiber and fiber-degrading enzymes in pig feed are known to affect expression of cytokines in the gut [74]. They found different effects of interleukins in porcine and murine enteroids, which shows the importance of using species-specific in vitro models for the target animal species. Additionally, Olayanju et al. argued that the use of porcine intestinal organoids have great potential in human biomedical research, for drug screening and biomarker discovery, as, pigs are closer to humans in anatomy and physiology than rodents [75]. In biomedical research on epithelial injury or diseases, porcine organoids may offer possibilities, particularly in situations where collection of tissue samples in humans from the pathogenic site for producing organoids may be too invasive or might induce pathology. Towards this, Von Furstenberg et al. developed an organoid model for porcine esophageal submucosal glands (ESMGs) and showed that the organoid model could be used to study differentiation into squamous versus columnar epithelium, and the mechanisms of ESMG proliferation and regeneration of injured epithelium [58]. Likewise, several studies aimed at providing an animal (porcine) organoid model for (human) biomedical research on small intestinal stem cell physiology and epithelial regeneration in short bowel syndrome, ischemic injury, and other conditions related to intestine [53, 54, 76]. Further, Adegbola et al. derived organoids from the anorectal epithelium to study etiology of, and therapies for, perianal Crohn’s fistulae [57]. Zhu et al. used porcine enteroids to study effects of (dietary) glutamate on pathways of importance for intestinal stem cell biology and intestinal epithelial proliferation [77]. Recently, Engevik et al. produced enteroids from genetically modified pigs to study microvillus inclusion disease, a rare genetic human disease of the intestine, characterized by chronic, watery, life-threatening diarrhea in infants [78].

In the area of pig nutrition, Wang et al. used pig enteroids as in vitro model, showing that vitamin A regulates the “stemness” of intestinal stem cells [79]. In other studies, porcine enteroids were used to demonstrate the impact of the food/feed-associated mycotoxin, deoxynivalenol [80] or the dietary amino acid l-Glutamate on intestinal stem cell activity, in particular by effects on molecular pathways that are essential for intestinal homeostasis and functionality [77, 81].

#### Chicken

Chicken intestinal organoids (small intestine, cecum) can be readily derived from sampled intestinal tissue following methods similar to those used for enteroids of pig and other species. Tissues were obtained from either pre-hatch chicklets [82–85], day-old male broiler chicks [86], young (2–3 week post-hatch) chicks [85, 87], or from adult chicken carcasses [55, 87].

Reverse transcriptase-polymerase chain reaction (RT-PCR), immunoblot analysis, and immunofluorescence microscopy indicated that the chicken intestinal organoids expressed markers for crypt stem cells, and for proliferating, differentiating, and mature enterocytes [82, 85], as well as goblet cells, enterochromaffin-like-cells’, and “Paneth-like” cells [86].

These studies give a clear outlook on applications of organoids, e.g. research on avian intestine physiology, drugs and feed absorption, interaction with microorganisms, and gut immunity [82, 85]. Pierzchalska et al. applied organoid cultures to study interactions of the intestinal epithelium with the probiotic *Lactobacillus acidophilus*, as well as ligands for toll-like receptors (TLR) 2 and TLR 4 [83, 84]. They described how organoids in culture can migrate and fuse with each other thereby forming bigger size organoids, and how this is influenced by the TLR4 ligand LPS. These results show the potential of chicken organoids to study early gut development and maturation as well as the interaction of the intestine with probiotic bacteria and with pathogenic and commensal microbiota, which is important for gut health and homeostasis, feed efficiency, and productivity.

#### Cattle

In several recent studies, bovine enteroids and colonoids have been generated (Table 2), using similar methods as described for other species. Like in other species, the bovine enteroids and colonoids could be maintained in culture for a long time [55, 88]. Bovine enteroids have been characterized by microscopy and histology, 5-Ethynyl-2′-deoxyuridine (EdU) staining for proliferative activity [55], and transcriptome analysis [89]. It was shown that enteroids can be cryopreserved and thawed to start continued culture for at least several passages [56, 89]. Transcriptome analysis at various time points (passages) confirmed long-term stability of the organoid cultures [89]. As mentioned for the pig, also cattle enteroids were used to study the interaction with pathogens *Toxoplasma gondii* and *Salmonella typhimurium* [56].

#### Sheep

Powell et al. showed that sheep enteroids can be readily derived from terminal ileum tissue, using similar methods as for example used in pigs [55]. In fact, they showed that these methods can be applied with minor modifications to a range of farm and companion animals including cat, dog, cow, horse, pig, sheep and chicken. The sheep enteroids were maintained in culture for a very long time, i.e. 239 days or 66 passages. Histology and transcriptome analysis confirmed that the sheep enteroids contained the principle cell types of the intestine epithelium which include absorptive enterocytes and stem/progenitor cells.

Liu et al. have established sheep pancreatic duct organoids to investigate the role and mechanism of copper in the sheep pancreas [90]. They showed that moderate concentrations of copper promote sheep pancreatic duct organoids and detailed the molecular mechanism through which copper induces the cell proliferation in the organoids.

#### Dog

In several recent studies, organoids have been generated from tissues from various organs of the dog (Table 2). Powell et al. derived ileum enteroids that were maintained in culture for as long as 229 days or 64 passages [55]. Chandra and et al. obtained organoids from several duodenum, jejunum, ileum, and colon, which they maintained in culture for more than 20 passages [91]. Both groups demonstrated that the intestinal organoids could be cryopreserved, thawed and expanded, providing a banked resource for continued experimentation. Similar to enteroids in other species, such as the pig, intestinal organoids from dogs consisted of different cell populations of intestinal epithelium including stem/progenitor cells, absorptive enterocytes (having microvilli expressing brush-border enzymes), tuft cells (expressing SOX9 and DCLK1 genes), tight junctions and Paneth-like cells [91]. It is noteworthy that while Paneth cells are reported to be absent in dogs [92], however, Chandra and et al. demonstrated functionally equivalent cells (Paneth-like cells) in the dog intestinal epithelium and enteroids [91]. The same authors applied a number of functional assays that can demonstrate and quantify organoid epithelial functions, such as optical metabolic imaging, the cystic fibrosis transmembrane conductance regulator function assay, and an assay for the uptake of exosome-like vesicles (from the parasitic nematode *Ascaris suis*). Such assays may be used to “phenotype” performance and drug response of animal-specific dog enteroids, which might be relevant for both human and veterinary gastrointestinal research and “personalized” medicine.

Dog keratinocyte organoids were produced to address skin disorders and alopecia in dogs [93]. These organoids are generated from either micro-dissected hair follicles or interfollicular epidermis. They could be maintained in culture for several months, remaining phenotypically stable as characterized by gene expression analyses, microscopy, histology and protein expression analyses.

Dogs are often used as a model in human biomedicine research, as the dog for some diseases bridges the gap between (often used) rodent models and humans [94, 95]. Using both wild type dogs and dogs deficient in COMMD1, which is essential for liver copper homeostasis, Nantasanti et al. developed a dog liver organoid culture system to validate stem cell and gene therapy strategies to cure copper storage disease in human [96]. Liver organoids were derived from fresh explanted or dimethyl sulfoxide (DMSO)-frozen liver, or from biopsy samples (wedge, or “Tru-Cut” or fine-needle aspiration biopsies) [96]. Like enteroids of dog and other species, the dog liver organoid cultures were maintained for as long as 8 months (28 passages) and could be resumed after cryopreservation of organoids. Furthermore, karyotype analysis of organoids showed that most cells (> 85%) retained the normal chromosome number, even after 8 months of culture [96], reflecting long-term genetic stability of the organoids. Further, budding tubule-like kidney organoids could be grown on MG from dissociated adult canine kidney cells [97], showing high self-renewal capacity in long-term culture (> 13 months). In this study, the authors hypothesized that the remarkable self-renewal capacity and the differentiation towards tubular cells is due to induced STAT3 expression at high cell density in these cells.

In addition to organoids from healthy tissues, prostate cancer [98] and bladder cancer organoids [99] have been generated from cancerous cells in urine from dogs with prostate or bladder cancer, respectively. Both organoid models resembled histopathological characteristics and gene expression profiles of the original tissues, and could be useful tools to provide insights into cancer therapy in dogs and as a translational model in prostate and bladder cancers in human.

#### Cat

In cats, organoids have been generated successfully from intestine and liver (Table 2) using current organoid techniques (as described in Figure 2). Cat organoids have been used in biomedical research and/or in veterinary research for the species itself. Ileum tissue obtained from euthanized cats was used to generate enteroids that were maintained for 67 days or 18 passages in culture and could be cryopreserved [55]. Similar to the enteroids from other species, enteroids from cats formed budding structures with distinct regions of cell proliferation (shown by EdU staining) in areas exhibiting crypt-like budding and folding. The cat enteroids expressed crypt stem cell marker LGR5. But unlike enteroids in the other species, the mesenchymal marker vimentin was expressed quite strongly, which disappeared around passage 7–9, followed by cessation of expansion around passage 10 with further growth arrest around passage 13–18. This suggested that mesenchymal-like cell types are essential for cat enteroid growth. Recently, Tekes et al. reported to have generated colon-derived organoids and studied host–pathogen interactions and immune response against feline enteric coronavirus [100].

Cat liver organoids were generated from post-mortem liver samples which were maintained for 25 passages in culture condition [101]. Further, karyotyping analysis has shown 80–85% of the cell population in the liver organoid retaining the normal chromosome number (i.e. 38), when maintained up to 23 passages in culture, reflecting long-term genetic stability of the organoids. The liver organoids could be cryopreserved and formed organoids again upon thawing. Feline liver organoids expressed markers of adult stem cells (LGR5, PROM1, and BMI1); hepatic progenitor cells (KRT7, KRT19, and HNF1b); early hepatocytes (HNF4a and TBX3, ALB), and mature hepatocyte (PROX1, PC, HMGCL, TTR, FAH, and CYP3A132). Recently, in a study by the same research group using cat liver organoids, two potential drugs useful in the treatment of hepatic lipidosis in cats were recognized [102].

#### Horse

Enteroids have also been derived in the horse (Table 2), as an additional in vitro model for studying gastrointestinal developmental biology, interactions with nutrients or with (pathogenic) microbes. Enteroids were generated using intestinal samples from euthanized horses [55, 103]. Immunofluorescent antibody histological staining and PCR indicated the presence of stem cells, enteroendocrine cells, absorptive enterocytes, goblet cells, and Paneth cells [103]. A long term maintenance (168 days, 46 passages) of horse enteroids has been reported [55], and enteroids could be cryopreserved, thawed, and expanded [55, 103].

## Potential improvements of organoid culture systems in livestock and veterinary research

Organoids offer a great potential for livestock and veterinary research. There seems to be little restriction regarding the types of tissue/organ and species from which organoids could potentially be derived. This also applies to other farmed animal species not covered in this review, such as rabbit [104] and fish species, e.g. rainbow trout [105]. However, to increase the scope of (large scale) application of organoids several challenges need to be addressed.

### High throughput, low cost, reproducible organoid platforms

Large scale applications of organoids would require reproducible, accurate, low-cost and high-throughput organoid platforms. Reproducibility may be improved by using well standardized protocols and more defined medium ingredients. Purified lyophilized (commercially available) niche factors appear to provide better defined and more constant quality than “conditioned medium”, i.e. medium from cultures of recombinant cells expressing the required niche factors Noggin, R-spondin, and WNT3A. The ECM, MG, with its complex, poorly defined and variable composition may also contribute to variation in the physical and biochemical culture conditions [106, 107]. Furthermore, the niche factors and MG contribute substantially to the high costs of organoid culture. For the culture of mouse intestinal organoids it was shown that epidermal growth factor can be replaced by lysophosphatidic acid [112]. Similarly, for the culture of chicken intestinal organoids, the expensive agonists R-spondin 1 and Noggin may be replaced by prostaglandin E2 [92]. As an alternative for MG, more defined and less costly biomaterials have been studied, like natural, synthetic and protein-engineered hydrogels [107–109].

The costs of organoid culture may also be reduced by using the “hanging drop culture”, which physically favors cell-to-cell interactions due to the lack of rigid support or solidified ECM scaffold [82]. This method features a lower concentration of MG (only 5%), resulting in an overall 70% lower expenditure of MG in comparison to the standard protocol [82]. In addition, this hanging drop culture method takes less time as it does not involve MG solidification. A recent review paper [38], describes how microfabrication methods and devices, such as lithography, microcontact printing, and microfluidic delivery systems, can help overcome current limitations of organoid culture regarding complexity, throughput, and reproducibility. In a study on bovine colonoids, Töpfer et al. introduced methodological advances such as extrusion bioprinting of colonoid fragments into multi-well plates as an alternative seeding and culture methodology, as well as in-plate cryopreservation as convenient alternative to conventional in-vial cryopreservation to enable a “plug and play” format for cell-based bio-efficacy and biosafety testing [88]. This and other cost and time effective findings may contribute to producing high throughput organoid platforms from various tissue/organs from livestock and companion animals.

### 2D organoid platforms

To enable access to the apical membrane of epithelial organoids, which is necessary to study e.g. interaction with microbes or transport of nutrients, 2D organoid models have been developed [41–43, 110–112]. It is necessary for farm animals that such 2D models are developed and validated. Recently, for pig intestinal organoids such 2D cultures on Transwell membranes have been established and used [43]. These 2D organoids are amenable for high-throughput systems e.g. measuring transcriptome response to variables of interests, including interactions with feed ingredients, drugs or pathogenic microorganisms [62, 72]. Furthermore, 2D intestinal organoid models allow electrophysiological studies using Ussing chambers as well as measurement of trans-epithelial permeability and electrical resistance as read-outs for intestinal (organoid) function which are relevant for studying “transport capacity” of the intestine. Such abilities make the 2D intestinal organoid model a powerful and sophisticated experimental model of mammalian biology for studying complex interactions occurring in the intestinal lumen. However, 2D models can also have disadvantages compared to 3D models. The 2D monolayers can contain all cell lineages found in ASC-derived organoids [43], but in other studies (reviewed in [113]) it has also been reported that some cell types present in 3D organoids may not be represented in 2D cultures. Moreover, the propagation from 2D to 2D can only be accomplished for a limited number of iterations [114].

### Collection of tissue samples; biopsies

Organoids in livestock and companion animals have only been produced from “parent” tissues (containing ASCs) collected from abattoir or euthanized animals. If animals need to be sacrificed specifically for this purpose, this would not comply with the 3R concept-Replacement, Reduction and Refinement. Also, the sacrifice of animals would not be desired by livestock breeders if the animals belong to their valued nucleus breeding population nor by animal owners in case of companion animals. Enabling the collection of tissue samples by biopsy, a common practice to collect tissue from human, can be a solution to collect “parent” tissue to culture organoids from livestock and companion animals, provided this can be achieved with minimal discomfort for the animals. A limited number of research articles reported collection of biopsies for the production of intestinal organoids in pigs [54, 115]. We have recently carried out a pilot study with the aim of using biopsy materials to culture intestinal organoids in pigs (unpublished). With the aid of colonoscopy, we collected sufficient biopsied tissue samples and successfully produced colonoids from these samples. Similarly, biopsy techniques (including needle biopsy [96]) may be used for obtaining tissue samples to produce organoids from other organs including gastrointestinal-, respiratory-, urinary-, and reproductive- tracts, as well as liver, mammary gland, fat and muscle, collected from livestock and companion animals. However, if this would be applied, utmost care must be given to minimizing discomfort or pain. When tissues from several organs are to be taken it may most likely require the sacrifice of an animal. The possibility of re-use of organoids (especially generated in a serum-free organoid culture system) stored in a biorepository would be in line with the 3Rs. Additionally, interesting non-invasive approaches seem to be possible in specific cases. For example from urine, human kidney tubuloids [116] and dog prostate cancer, organoids [98] have been cultured and expanded. It would be interesting to explore if similar approaches would be feasible for producing udder organoids from (stem) cells in milk for dairy cattle.

### Validation

It is important to validate organoids as a model for the organ or tissue of origin, or even as proxy for in vivo performance characteristics and animal (genetic) differences thereof. This would involve comparing characteristics and performance of tissue from animals, or even of intact animals, with characteristics and performance of organoids derived from these tissues. There have been only a few studies in that regard in livestock and companion animal species. In the pig, Van der Hee et al., have compared the transcriptomes profiles of jejunal organoids, the corresponding jejunum mucosa tissue from which the organoids were derived, and IPEC-J2 cells [60]. They found that the set of genes expressed in the organoids closely resembled that of the tissue of origin, including small intestine-specific genes, most of which were not expressed in the IPEC-J2 cells. Regarding nutrient uptake studies, mouse and human organoid models have been validated in a qualitative sense as valuable tool [117]. In a quantitative sense, studies would be needed to compare both expression of transporter proteins and actual nutrient transport kinetics with uptake kinetics of intestinal tissue measured in Ussing chambers [118] or with uptake kinetics measured in live (farm) animals [119]. Preferably, organoids derived from animals that have a clear contrast in a trait such as disease resistance or feed efficiency could be compared. For instance we are currently looking at genome wide gene expression of intestinal organoids derived from pigs with different feed efficiency [59, 120].

### Development of more complex systems

The in vivo intestine contains, in addition to the epithelium, a complex immune and neural system. In the in vitro organoid system, this complex immune and neural system is lacking, which reduces its ability to study interactions between these (sub)systems. Co-culturing of organoids with immune or neural cells and providing tissue specific biochemical cues resembling the in vivo condition could in part enable the study of such interactions. However, co-culture of various cell types in an organoid system has not been reported for livestock and companion animals yet. Techniques such as 3D bioprinting (e.g. [38, 88]) for seeding culture devices may enable co-culturing of various cell types with defined spatial positioning to generate more complex organoid systems that may better mimic the in vivo host physiology.

## Potential uses of organoids in livestock and veterinary research

### Fundamental biology and pathology

The respiratory, gastrointestinal, urogenital, and mammary epithelia, along with the skin, are the most important sites of contact with the outside environment, with intimate contacts with the respective microbiomes of these organs, including potential pathogens. While interaction with microbes has mostly been studied in organoid models of intestinal epithelia of different species [55, 56, 59, 61–64, 71–73, 83, 84, 91, 100, 103], similar studies could be done in organoid models as have been done in human and rodents for the lung [121] and the mammary gland [122]. In the gut, the epithelium is constantly screening the contents of the intestinal lumen [123, 124]. Intestinal immune signaling can be triggered by nutrients, the digestion products from feed, and the intestinal microbiome, including pathobionts. The signaling modulates the innate and the adaptive immune system while trying to maintain a balance between pro- and anti-inflammatory conditions to preserve gut health and homeostasis [73]. Some aspects of this sensing and signaling may be studied in intestinal organoids, for instance the production of, and response to immune signaling molecules such as cytokines and/or enteroendocrine hormones [74, 125, 126].

Research on primary epithelial samples form human fetal and pediatric samples has shown changes in DNA methylation associated with different expression programmes [127]. These changes are likely to be mediated by the exposure to environmental factors changing during early life. Epigenetic modifications have also been reported in colon cancer, for example hypermethylation of the promoters of tumour suppressor genes influencing tumour growth [128]. Research emerging from bio-medical domain provide evidences that organoids offer possibility to study epigenetics in health and diseases [129–131]. In livestock or veterinary research, organoids (of relevant tissue) from livestock or companion animal species could be employed as a tool to study epigenetics, that may generate new knowledge towards various aspects of early life programming or imprinting in livestock species [132, 133] acquired via the nutritional or managemental practices adopted in animal husbandry.

### Nutritional research

In the area of animal nutrition, intestinal organoids may be used to study the gastro-intestinal epithelial response to feed ingredients (as has been done with mouse organoids [112]). Performing such studies could aid nutritionist to determine “non-strict-nutritional” properties e.g. anti-oxidative and oxidative effects, immune responses and signaling, arising from digestion of proteins [134], carbohydrate [135] or other dietary components [136].

### Breeding

A wholly different concept of implementing organoids is their use for phenotyping animals for the purpose of selective breeding. Here, the goal is not necessarily to advance our understanding of animal traits, or to understand specific mechanisms, but simply to characterize (“phenotype”) organoids of as many as possible animals with known genotypes. This could be an important tool to further improve production performance in livestock. For instance, in finding quantitative trait loci (QTLs) and potentially causative genes for specific traits by searching for genome wide associations between specific phenotypes and genomic information [25, 26, 137]. Organoids may also contribute to genomic selection, in which the genetic merit of breeding stock is not directly obtained by phenotyping but is rather inferred from genomic information for which the relationship with the phenotype is established in a “training” population of phenotyped animals [138, 139]. It can thus be envisaged that a repository of organoids is established from animals representing the training population. In vitro phenotyping of these organoids could then be used instead of, or in addition to, “normal” phenotyping of these animals, as this would provide phenotypic information on more defined underlying (cellular, molecular) aspects of the traits of interest. Livestock improvement currently has a focus on traits related to animal health and resilience, animal welfare, and feed efficiency [140, 141]. Animals may have genotypic differences regarding their interaction with microbes and this may manifest itself on a cellular or molecular phenotypic level. Organoids representing the relevant epithelia may be suited for measuring certain aspects of the interaction with microbes or the transport of nutrients [59, 120]. Large-scale application of organoids in breeding programs would require a high-throughput and low-cost organoid platform allowing standardized, reproducible and accurate measurements of in vitro performance of epithelial functions. Further, if tissues for deriving organoids could be obtained from biopsies from live animals (discussed in Sect. 4), this would have the advantage that after in vitro phenotyping the high merit animals are still available as breeding stock.

### Routine testing

Lastly, organoids may be used to constitute a routine testing platform. Here, the goal is not necessarily to understand mechanisms or develop medicines or diets, but rather to apply (validated) relationships. For instance, organoid platforms may be made available for testing of effectiveness and side effects of (veterinary) drugs. Large-scale and high-throughput organoid platforms could also be made available for routine toxicologic testing [142], or for routine quality testing of diet or feed ingredients [80, 143]. Also, for human health, human (or animal) organoid platforms may be used for routine testing of products for human consumption [144]. Especially in such routine applications, organoids and other in vitro or ex vivo models can contribute to a reduction of animal studies, in line with the principles of the 3Rs.

## Conclusion

Organoids can be important in vitro research tools, in fundamental, applied, or routine aspects of veterinary and animal production sciences and may complement and partly replace animal studies. This would require more research, especially regarding organoids of other organs, as the majority of studies have been on intestinal organoids. Organoids have distinctive advantages over other in vitro models, as they better recapitulate structure and function of tissues. Compared to intact organs they are strongly reduced models, which may be an advantage for studies on specific mechanisms, but also confers clear limitations to the model. Organoids thus provide a well-defined, accessible research model that may be used to obtain phenotypic information on defined underlying cellular and molecular aspects of important complex traits such as feed efficiency and disease resistance. Thus, organoids can be of great value in livestock and veterinary research.

## Data Availability

Not applicable

## Abbreviations

2D: Two-dimensional
3D: Three-dimensional
3Rs: Replacement, reduction and refinement
ASCs: Adult stem cells
CRIPR-Cas9: Clustered regularly interspaced short palindromic repeats and CRISPR-associated protein 9
DMSO: Dimethyl sulfoxide
ECM: Extracellular matrix
EdU: Ethynyl-2′-deoxyuridine
ESCs: Embryonic stem cells
ESMG: Esophageal submucosal glands
HMI: Host-microbe interaction
IPEC: Intestinal porcine epithelial cell line
iPSCs: Induced Pluripotent stem cells
LPS: Lipopolysaccharide
PDCoV: Porcine delta corona virus
PEDV: Porcine epidemic diarrhea virus
PSCs: Pluripotent stem cells
QTLs: Quantitative trait loci
TLR: Toll-like receptors
